# Solitary bone plasmacytoma of spine with involvement of adjacent disc space: A case report

**DOI:** 10.1097/MD.0000000000027288

**Published:** 2021-09-17

**Authors:** Hongdong Tan, Jia Gu, Liang Xu, Gang Sun

**Affiliations:** a Department of Orthopedics, Shandong public Health Clinical Center (Shandong Province Chest Hospital), Shandong Province, P.R. China; b Department of pathology, the 960th Hospital of Joint Logistics Support Force of PLA, Shandong Province, P. R. China; c Department of Radiology, the 960^th^ Hospital of Joint Logistics Support Force of PLA, Shandong Province, P.R. China.

**Keywords:** intervertebral disc, solitary plasmacytoma, spine

## Abstract

**Rationale:**

Solitary bone plasmacytoma (SBP) is a rare manifestation of plasma cell tumor. Although axial skeleton is the most frequently affected site of SBP, adjacent disc space involvement is rare. Herein we report a case of SBP in thoracic vertebra with involvement of adjacent disc space.

**Patient concerns:**

A 57-year-old male presented with a 2-year history of intermittent back pain with activity. The patient's back pain intensity with activity was a score of 5 of the 10-point visual analog scale).

**Diagnoses and intervention:**

The patient underwent a posterior fusion procedure from T6 to T10, and an open biopsy of the vertebral lesion confirmed that final diagnosis of SBP. The patient received postoperative radiotherapy with a total of 4000 Gy to the T7–T9 vertebral levels over a 20-day period.

**Outcomes:**

Following radiotherapy, the patient's pain intensity was reduced to the visual analog scale score of 1 at the 6-month follow-up.

**Lessons:**

SBP lacks typical clinical symptoms, and the accurate diagnosis before clinical intervention remains challenging. Due to the disc involvement, SBP often manifests as spinal infection. Hence, differential diagnosis in spinal lesions involving the disc should include SBP.

## 1 Introduction

The most common complaint of patients with spinal disease is back pain, which has a series of underlying causes such as infection and neoplasm. Differentiating these causes of back pain is important to diagnosis and medical management. This case report described a patient presented with back pain caused by a solitary bone plasmacytoma (SBP). SBP is characterized by a localized proliferation of neoplastic plasma cells in the absence of significant bone marrow plasma cell infiltration, which occurs primarily in red marrow-containing bones such as vertebrae, femurs, pelvis, and ribs.^[1–3]^ To the best of our knowledge, there is only 1 report in the literature describing BP in vertebra with involvement of adjacent disc space.^[4]^ The presentation of vertebral lesion with adjacent disc space involvement usually indicates spinal infection, whereas the possibility of an SBP involvement is rarely considered. We here report a case of SBP in the thoracic spine involving an adjacent disc space, and its differential diagnosis from spinal infection.

## 2 Ethical statement and consent

The Institutional Review Board and Ethic Committee of Shandong Province Chest Hospital and the 960th Hospital of Joint Logistics Support Force of PLA approved the proposal of this investigation.

Written informed consent was obtained from the patient for this case report to be published (including images, case history and data).

## 3 Case presentation

**Figure 1:**
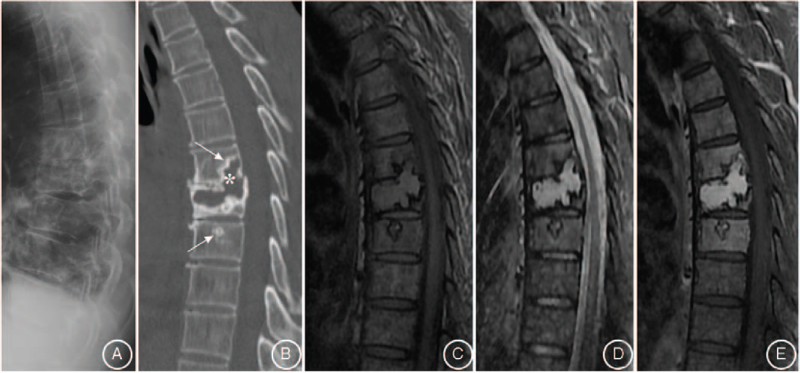
Lateral spine radiograph demonstrated osteolytic destruction of T7 and T8 vertebral body with intervertebral space stenosis (A). Mid-sagittal CT of thoracic spine suggested slight expansile osteolytic destruction in T8 vertebral body with sclerosis margin, extension to T7 and T9 vertebral body (arrows) and adjacent disk-space destruction (asterisk) (B). The corresponding thoracic spine MRI revealed the lesion exhibiting mildly isointense on T1 weighted image (C), hyperintense on T2 weighted image (D), and intense homogeneous enhancement on T1-weighted post contrast image (E).

**Figure 2:**
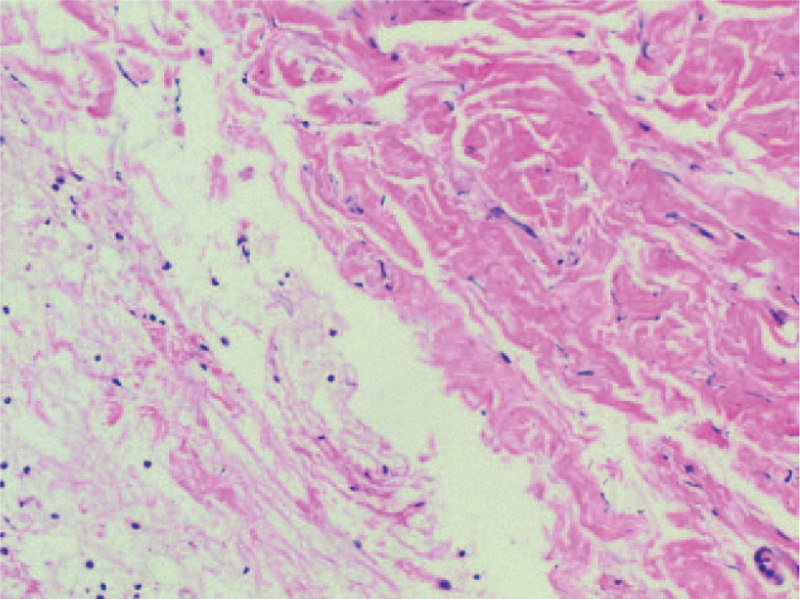
Hematoxylin and eosin (H&E) stained biopsy specimen showed a small number of fragments of bone trabeculae and denatured collagenous fibers with inflammatory exudates (100×).

**Figure 3:**
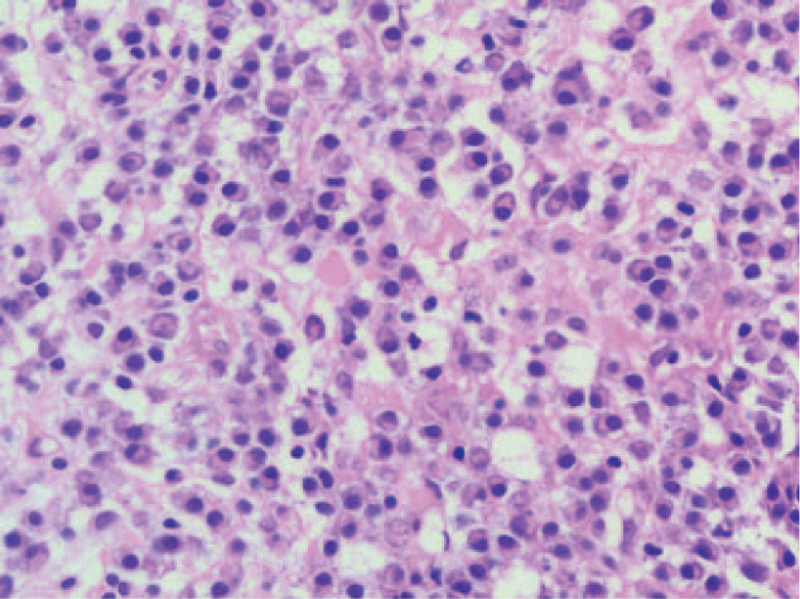
Hematoxylin andeosin (H&E) stained section showed a diffuse monotonous infiltrate of plasma cells (200×).

**Figure 4:**
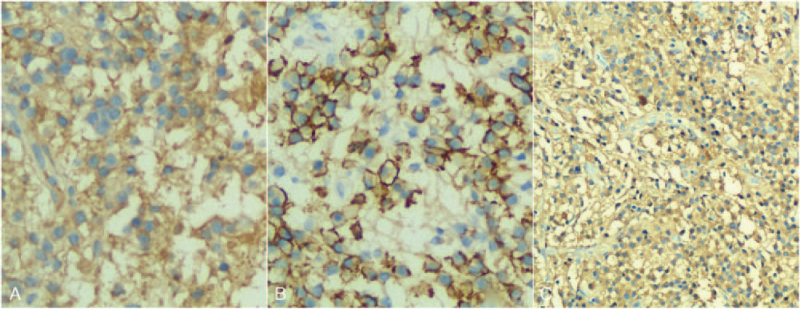
Immunohistochemical markers were positive for CD38 (A, 200×), CD138 (B, 200×). Lambda was positive (C, 100×). Kappa was negative (not shown).

A 57-year-old male was admitted with a chief complaint of intermittent pain in the back for the past 2 years. The pain was nonradiating and not responding to conservative medical management. The patient's pain intensity score was 5 on a 10-point visual analog scale. He had no symptom of sensorimotor neuropathy. The severity of the neurologic deficit was defined as “E” according to the American Spinal Injury Association Impairment Scale. His medical history and findings from physical examinations were unremarkable. His routine blood tests were within normal range and the urine analysis showed normal values. Old tuberculin test and brucellosis agglutination test were negative. His lateral spine radiograph showed osteolytic destruction from T7 to T8 vertebrae with intervertebral space stenosis. Computed tomography (CT) revealed slight expansile osteolytic destruction at T8 with sclerosis margin, extended to the T7 and T9 and adjacent disc space destruction. Magnetic resonance imaging (MRI) showed the lesion centered in the T8 vertebral body extending to T7 and T9 with disc space destruction. The lesion was mildly isointense on T1-weighted images, hyperintense on T2-weighted images, and homogeneously enhanced on T1-weighted post contrast images (Fig. 1). There is no evidence of spinal stenosis. Transpedicular needle biopsy of T8 lesion showed fragments of bone trabeculae and denatured collagenous fibers with inflammatory exudates (Fig. 2). Concerning the spinal instability, surgery was scheduled after discussion at multidisciplinary consulting meeting despite the lack of a definitive preoperative diagnosis. Under general anesthesia, the patient underwent a posterior fusion procedure from T6 to T10 vertebrae, and an open biopsy at the T8 lesion site. The open biopsy samples revealed a large amount of plasma cell infiltration (Fig. 3). Immunohistochemical studies showed sheets of lambda positive CD38, CD138 positive plasma cells (Fig. 4). The plasma cells showed expression of IgA but were negative for IgG, IgM, and CD20.

**Figure 5:**
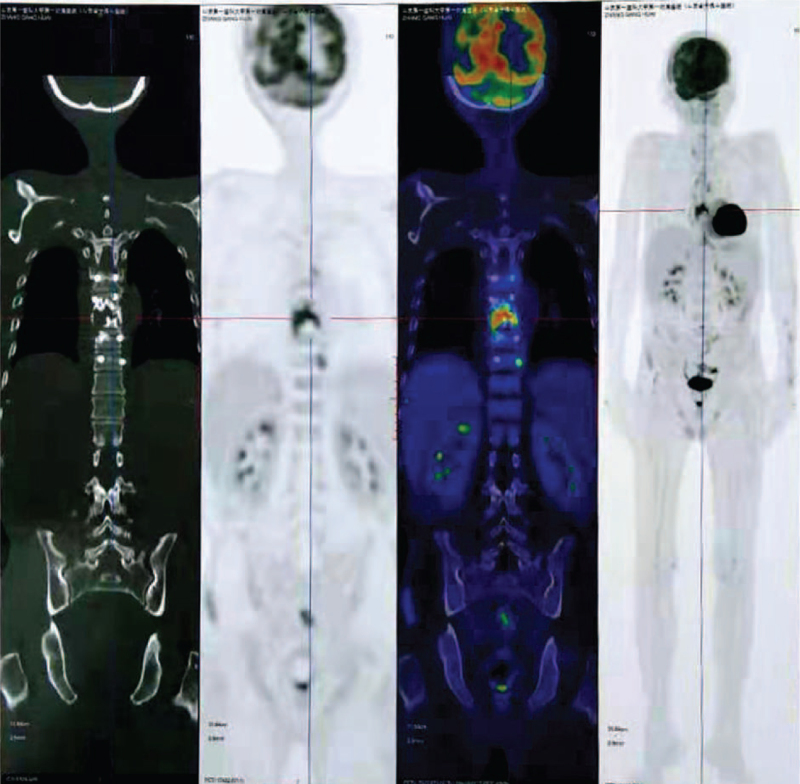
PET-CT scan showed the hypermetabolic lesion in T7 to T9.

**Figure 6:**
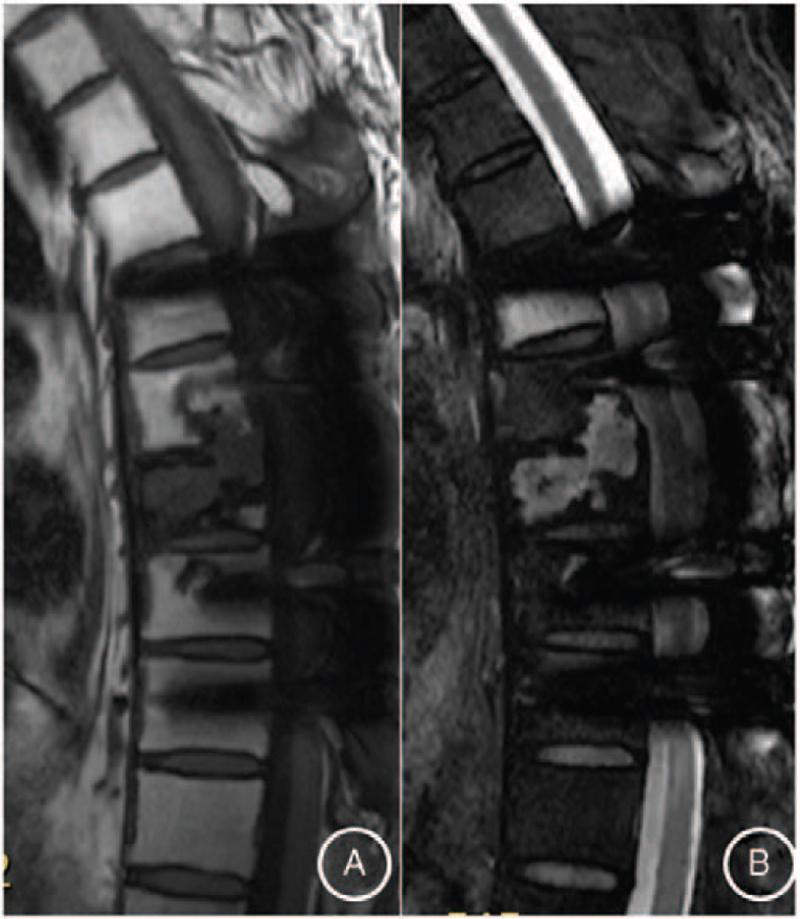
Thoracic spine magnetic resonance imaging at 6 months after radiotherapy showed the lesion was smaller compared to the pre-treatment imaging (A). The lesion exhibiting mildly isointense on T1 weighted image (B). Hyperintense on T2-weighted image.

Postoperative fluorodeoxyglucose-positron emission tomography/CT showed that the T7-T9 lesion was fluorodeoxyglucose avid with standard uptake value of 10.9, and no other suspicious hypermetabolic lesions (Fig. 5). Based on the pathological results, the patient was diagnosed as SBP with minimal marrow involvement. Complete skeletal radiographs revealed no evidence of other osteolytic lesions. In addition, laboratory studies, including creatinine, hemoglobin, calcium, and 24-hour urine protein electrophoresis showed normal values. Forty-five days after the operation, the patient received radiotherapy with a total of 4000 Gy to T7 to T9 vertebrae over a 20-day period. Following radiotherapy, the patient's pain intensity was reduced to the visual analog scale score of 1 at the 6-month follow-up. Furthermore, the lesion size became smaller on the thoracic spine MRI at the 6 month's follow-up (Fig. 6).

## 4 Discussion

SBP is a rare hematologic malignant disease, which is defined by the presence of a single osteolytic lesion due to monoclonal plasma cell infiltration, with or without soft-tissue extension.^[2]^ The median age at the diagnosis is 55 years and males are more frequently affected than females. The lesion is often present for many years as a single lesion; however, it can eventually progress to multiple myeloma (MM).^[5]^ Currently, regional radiotherapy continues to be the recommended treatment for SBP at a dose of 40 to 50 Gy over approximately 4 weeks.^[2]^ The response rate to radiotherapy has been shown to be 94%. The adjuvant chemotherapy did not affect the incidence of progression of SBP to MM, but it did delay the progression from 29 to 59 months. Ultimately, 53% of patients with SBP develop MM within 17 years of treatment, and 14% develop local recurrence within 5 years of treatment.^[2,6,7]^

The most common site of SBP is in the vertebrae, especially in the thoracic vertebrae. Back pain is a common clinical feature due to lesion compression.^[8,9]^ Diagnostic criteria of a SBP include pathological proven solitary lesion, normal bone marrow with no evidence of clonal plasma cells, normal skeletal survey and MRI (or CT) of spine and pelvis (except for the primary solitary lesion), and absence of end-organ damage.^[1]^ Our case meets all these criteria for the diagnosis of SBP. Several studies have reported that patients complained back pain on average 8 months before the diagnosis of SBP. In those cases, patients visited various health care services on multiple occasions leading to the SBP diagnoses; some even received incorrect diagnoses.^[10,11]^ Imaging studies play an important role in the diagnosis, treatment, and prognosis. However, imaging studies may not always provide a conclusive diagnosis. SBP has an osteolytic appearance on plain radiographs, and an expansile osteolytic lesion with marked enhancement on CT or MRI.^[2]^ SBP of spine could show curved coarse trabeculae with hypertrophic sclerosis, forming “mini brain sign” which may have certain characteristics. This appearance may be correlated with the less aggressive nature of SBP comparing with other malignant tumors that aggressively destroy the bone with no radiological evidence of bone repair features such as sclerosis and thickening.^[12,13]^ In our case, we could not observe this similar imaging feature. To the best of our knowledge, most studies did not report the imaging presentation of SBP involving adjacent disc space. In fact, only in 1 case of SBP, involvement of the adjacent disc space (by direct extension) was reported. Afonso et al reported a 41-year-old woman with SBP, her spine CT revealed areas of destruction in both the vertebrae and discs between T12 and L2.^[4]^ That case report mainly focused on CT and MRI findings, and did not offer differential diagnosis of spinal neoplasms and infections in the involved vertebrae and associated disc spaces.

Usually, spinal lesions associated with a poorly defined vertebral body endplate, involvement of intervertebral disc space, presence of paravertebral abscesses, and involvement of two contiguous vertebral bodies are suggestive of spinal infection. On the contrary, spinal lesions associated with a well-preserved disc space, destroyed vertebral bodies with solid extraosseous soft tissue, skip or nonconsecutive multifocal involvement of spine are suggestive of spinal neoplasm.^[14,15]^ However, 1 study found that 2% malignant spinal lesions involved vertebral discs.^[16]^ SBP with adjacent disc space destruction of the patient may be due to aggressive traits of primary plasmacytoma infiltrating and destructing adjacent bones, muscles, fats, and vascular encasements.^[17,18]^ In spinal lesions involving adjacent disc spaces, the differential diagnosis should include neoplasm and bacterial infection tests. In some conditions of spinal infection, patients can be afebrile with nonspecific symptoms, and imaging findings can be nonspecific or atypical. Laboratory examinations including white blood cell count, erythrocyte sedimentation rate and C-reactive protein are inconclusive^[19,20]^ and biopsy is warranted to establish diagnosis. However, a study reported that the presumed etiology in 18% of the cases was not confirmed on pathological examination.^[21]^ A recent meta-analysis reported the sensitivity of CT-guided percutaneous needle aspiration biopsy was 52.2% (95% confidence interval, 45.8–58.5) for the diagnosis of spinal infections.^[22]^ The presentation of disc involvement is a nonspecific feature of spinal infection, and can be SBP, as is shown in our case. Hence, differential diagnosis between spinal neoplasm and infection could be more difficult and complicated. The diagnostic approach for the patients of spinal lesions with disc involvement should include a complete medical history, physical examination, and necessary laboratory examinations and imaging evaluations during which possible risk factors for infections and neoplasm must be investigated and identified. It is important to recognize the imaging findings as the signs of spinal neoplasm and infection, and a short-term follow-up imaging examination should be performed to detect any changes. In some cases, repeat or open biopsy may be required as it is the only reliable method to distinguish neoplasm versus infection.

## 5 Conclusions

In conclusion, our case illustrated atypical imaging features of SBP with disc involvement. The imaging feature of disc involvement may be difficult to differentiate from spinal infection. Importantly, we conclude that differential diagnosis of spinal SBP with disc involvement and spinal infection relies mainly on the combined application of imaging examination and its correlation with clinical history and laboratory tests. Due to limited literature available, our case report may help to recognize this rare sign of SBP with disc involvement, thus avoiding misdiagnosis and mistreatment.

## Author contributions

**Conceptualization:** Gang Sun.

**Data curation:** Hongdong Tan, Jia Gu, Liang Xu.

**Investigation:** Jia Gu, Liang Xu.

**Methodology:** Hongdong Tan, Jia Gu.

**Supervision:** Gang Sun.

**Writing – original draft:** Hongdong Tan, Gang Sun.

**Writing – review & editing:** Hongdong Tan, Gang Sun.

Hongdong Tan: data collection and analysis, MRI and manuscript writing; Liang Xu: data curation; Jia Gu: pathology; Gang Sun: conception and design and manuscript writing.
